# Differential Regulation of Human Surfactant Protein A Genes, SFTPA1 and SFTPA2, and Their Corresponding Variants

**DOI:** 10.3389/fimmu.2021.766719

**Published:** 2021-11-30

**Authors:** Joanna Floros, Nikolaos Tsotakos

**Affiliations:** ^1^ Department of Pediatrics, The Pennsylvania State University College of Medicine, Hershey, PA, United States; ^2^ Department of Obstetrics and Gynecology, The Pennsylvania State University College of Medicine, Hershey, PA, United States; ^3^ School of Science, Engineering, and Technology, The Pennsylvania State University - Harrisburg, Middletown, PA, United States

**Keywords:** transcription regulation, variants, posttranscriptional regulation, promoter, surfactant protein A (SP-A)

## Abstract

The human *SFTPA1* and *SFTPA2* genes encode the surfactant protein A1 (SP-A1) and SP-A2, respectively, and they have been identified with significant genetic and epigenetic variability including sequence, deletion/insertions, and splice variants. The surfactant proteins, SP-A1 and SP-A2, and their corresponding variants play important roles in several processes of innate immunity as well in surfactant-related functions as reviewed elsewhere [1]. The levels of SP-A have been shown to differ among individuals both under baseline conditions and in response to various agents or disease states. Moreover, a number of agents have been shown to differentially regulate *SFTPA1* and *SFTPA2* transcripts. The focus in this review is on the differential regulation of *SFTPA1* and *SFTPA2* with primary focus on the role of 5′ and 3′ untranslated regions (UTRs) and flanking sequences on this differential regulation as well molecules that may mediate the differential regulation.

## 1 Introduction

As a way of background, pulmonary surfactant, a lipoprotein complex, is essential for life. It prevents alveolar lung collapse by lowering the surface tension at the air-liquid interface of the lung alveolus. Lung alveoli are the distal airspaces in the lung, lined by epithelial Type I and Type II cells. Under normal conditions macrophages are the only immune cells present in the alveolar space. The alveolus is covered by a thin liquid layer, called hypophase. Surfactant is found at the surface of the hypophase, i.e., at the air-liquid interface, as well as in the hypophase as a surfactant reservoir. Through its ability to maintain lung alveolar stability, surfactant enables the lung to carry out its key function of O_2_/CO_2_ exchange. Deficiency of surfactant in prematurely born infants and dysfunction of surfactant in adults can potentially lead to serious breathing problems including death.

Pulmonary surfactant is composed of about 90% of lipids, primarily phospholipids and four non-serum proteins, the surfactant protein A (SP-A), SP-B, SP-C, and SP-D. SP-B and SP-C are hydrophobic proteins and are involved in activities that primarily affect the surfactant function. For example, surfactant is found in the form of a monomolecular surface film at the air-liquid interface of the alveolus, which is responsible for the reduction of surface tension and thus prevention of lung collapse, and it is also found as surfactant reservoir in the hypophase. The two surfactant compartments are interconnected. During a breath there is reorganization of surfactant layers and the hydrophobic proteins are key for surfactant multilayer connection and bringing lipids from the hypophase to the air-liquid interface (1, 2). SP-A and SP-D are hydrophilic proteins and both belong to the collectin family of proteins. These are primarily involved in innate immunity, regulation of inflammatory processes and may serve as a link to adaptive immunity. In addition, SP-A contributes to various aspects of surfactant structure such as in the formation of tubular myelin (an extracellular structural form of surfactant), and the reorganization of surfactant in the hypophase. SP-D may play a role in surfactant homeostasis (1).

SP-A was the only known surfactant protein at the early times of clinical surfactant replacement trials (3, 4). The success of a human clinical study in 1980 (5), where pulmonary surfactant derived from cow lung (a natural source) was used successfully to treat prematurely born babies at risk for respiratory problems, and the failure of previous studies where off the shelf lipids were used (6), raised interest in the study of SP-A and led to the discovery of the other surfactant proteins (7–15). Although the surfactant proteins in the early years were known by various names, a nomenclature, used today, was agreed upon soon after their discovery (16). SP-A, the focus in this review, in addition to its surfactant-related functions, plays a role in the lung innate immune response and regulation of inflammatory processes (1).

**Figure 1:**
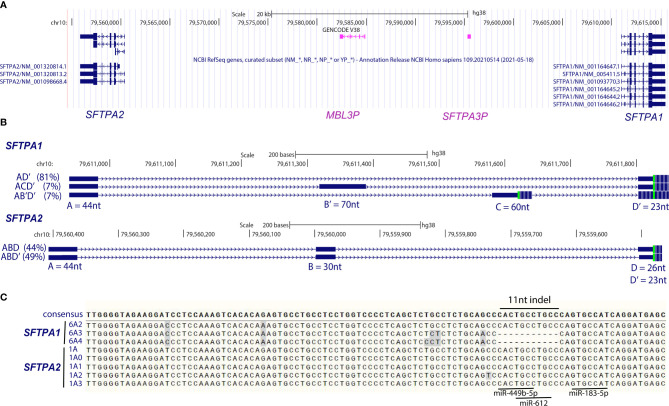
Human *SFTPA1* and *SFTPA2* genes and 5′-UTR and 3′-UTR variability. **(A)** The human *SFTPA1* and *SFTPA2* genes, encoding SP-A1 and SP-A2, respectively, are in opposite transcriptional orientation. The *SFTPA3P* is an SP-A pseudogene, and the *MBL3P* is the mannose-binding lectin pseudogene. The orientation shown is from the centromere (left) to telomere (right). **(B)** *SFTPA1* and *SFTPA2* 5′ UTR variability. The 5′-UTR consists of a number of untranslated regions as shown in blue boxes. These regions splice to form a number of transcripts with different 5′-UTR. The most common splice variants for *SFTPA1* and *SFTPA2* are shown and their relative presence in the general population is shown in parentheses. Regions A, C, and D can exhibit different start sites and region B exhibits different stop sites. For example, “A” is found in transcripts with a start site at A, A′, or A′′. The size of A is 44nt, of A′ is 40nt and of A′′ is 35nt. The C is 60nt long and the C’ is 63nt long. Region “B” on the other hand is found in transcripts, having the same start site but different end region. So B is 30nt long and B′ 70nt. The nucleotide size of each region is noted. In-frame start codons are indicated by a green vertical line. Adapted from (54). **(C)** The 3′-UTR has been identified with sequence variability as well as small insertions/deletions (indel). The 11-nt indel of the 3′-UTR is shown along with the seed regions for miR-449-b-5p, miR-612, and miR-183-5p, which have been shown to play a role in miRNA-mediated regulation. The figure was prepared from the UCSC Browser (hg38) and the alignment was performed by ClustalW.

Unlike rodents that have a single gene, humans (17, 18) and primates (19) have two genes, the result of gene duplication about 26.5 million years ago (19). The human *SFTPA* locus has been mapped at q22-q23 of chromosome 10 (20–22). This locus consists of two functional genes, *SFTPA1* and *SFTPA2*, in opposite transcriptional orientation with a pseudogene, *SFTPA3P* (23), in reverse orientation relative to *SFTPA1* at about 15kb away from the 5′ region of *SFTPA1* (21). The two genes are in linkage disequilibrium (24). The *SFTPA* locus also includes another pseudogene, the *MBL3P* (mannose binding lectin family member A3 pseudogene) (**Figure 1A**) (25). Although one study found the *SFTPA* locus close to the *MBP* locus (26), another study found that the *MBP* locus is located at a large distance, at about 25,000-35,000kb, from the *SFTPA* locus (21). Radiation hybrid mapping has placed *SFTPA2* and *SFTPD* (another surfactant protein gene) on the 5′region of *SFTPA1* at about 40 and 120 kb, respectively. Their orientation relative to the centromere is *SFTPD-SFTPA2-SFTPA3P-SFTPA1*-telomere. Although the evolutionary advantage of the *SFTPA* gene duplication has not been studied, one can only speculate. The SP-A protein is shown to be relatively conserved through the major vertebrate groups and it has been proposed that the surfactant system is an evolutionary prerequisite for air-breathing species (27). We postulate that the original role of SP-A was surfactant-related and at some time in evolution was “co-opted” to serve in host defense. Perhaps with the dual role, the *SFTPA* gene was subject to evolutionary selection that led to gene duplication. The available literature, as discussed elsewhere (1) indicates that for the most part both gene products carry similar functions but one seems to do a better job in host defense activities and the other in surfactant-related activities. Nonetheless, the functional complementation of the two protein products may mean that the gene duplication in the primate lineage was followed by subfunctionalization *via* selective pressure that keeps both genes functional (28). This is in contrast with another host defense gene, the mannose binding lectin (MBL), which underwent pseudogenization and lost its second functional gene in humans (29). At present, this hypothesis is simply speculative.

The *SFTPA1* and *SFTPA2* genes encode proteins that contain both collagenous and carbohydrate regions (22, 30) that places them in the family collagenous C-type lectins or collectins. SP-D, another surfactant protein encoded by the *SFTPD* gene, also contains both collagenous and carbohydrate regions and it is placed in the same family of proteins (31), along with the mannose binding protein (22). Collectins are soluble pattern recognition receptors and are part of the innate immune system (32–35). They bind various molecules on the surface of microorganisms, such as carbohydrate containing structures and lipids and may eliminate microorganisms employing various mechanisms. Collectins may also modulate regulation of inflammatory or allergic processes and the adaptive immune system. In addition, they play a role in the clearance of apoptotic cells (36).

The human surfactant protein A genes have been identified with extensive genetic and epigenetic variability in coding and non-coding regions and this variability has been associated with many pulmonary diseases (37–43). The impact of the coding genetic variability on function has been reviewed elsewhere (1). Briefly, the most frequently observed coding variants in the general population for each *SFTPA* gene are six (1A, 1A^0^, 1A^1^, 1A^2^, 1A^3^, 1A^5^) for SP-A2 and four (6A, 6A^2^, 6A^3^, 6A^4^) for SP-A1 (30, 44). However, the focus in the present review is primarily on the role of untranslated and flanking regions on the regulation of human *SFTPA* genes.

The 5′ flanking regions of the *SFTPA1* and *SFTPA2* genes share some conserved cis-acting regulatory elements with differing degrees of sequence conservation. Such elements have been shown to play a role in the differential regulation of the two genes, *via* certain transcription factors, such as NKX2.1/TTF-1 and NF-kB, or transcription coactivators such as the CBP/p300 factors. These may explain the differential regulation under different stimuli, such as in the presence of dexamethasone or cAMP analogs (45–47). In addition, epigenetic regulation can partially explain the differential regulation of the two genes. DNA methylation sites have been identified upstream of the transcription start site (TSS) of both *SFTPA1* and *SFTPA2* (48, 49), while histone modifications have been implicated in the regulation under certain stimuli (50).

The human surfactant protein is extensively co- and post translationally modified resulting in a large number of isoforms as shown by two-dimensional gel electrophoresis. Following the use of metabolic inhibitors and enzymes, this complex group of isoforms is reduced to a small number of isoforms that coincide with the isoforms of primary translation products of human lung RNA (51–53). The cloning of the genomic *SFTPA1* and *SFTPA2* sequences (17, 18) and of their cDNAs (52), enabled comparison of the two groups of sequences and this comparison revealed 5′-untranslated region (UTR) splice variability and 3′-UTR sequence variability in the *SFTPA1* and *SFTPA2* transcripts, as discussed below, as well as sequence variability within the coding region, but this has been reviewed elsewhere (1).

In humans, multiple transcripts have been identified that are due to 5′-UTR splice variability (54). Although in other species a single transcript (55) or more than one transcript (19, 27) have been identified, it is not known whether these are due to differences in splicing or polyadenylation. Karinch et al. (54), employing primer extension in an attempt to map the transcription start site of each human *SFTPA* gene and 5′RACE (rapid amplification of cDNA ends) to fully characterize their transcripts, using RNA from two unrelated individuals, discovered an extensive 5′-UTR splice variability as shown in **Figure 1B** (upper panel). Exon C′, not shown in **Figure 1**, and a similar 5′-UTR splice variability was also described by McCormick et al. with a different terminology (56). In this review, we use the Karinch terminology because this has been largely used in subsequent publications. *SFTPA1* transcripts appear to use A, A′ and A′′ transcription start sites of “exon A” with equal frequency but the *SFTPA2* transcripts use only the A site of “exon A”. The significance of this (if any) has not been addressed further (54).

The major 5′-UTR variants for *SFTPA2*, ABD and ABD′, are distinguished from the major *SFTPA1* 5′-UTR variant by the inclusion of exon B (eB). The difference between D and D′ is 3 nucleotides with the D′ having the additional 3 nucleotides, the result of a splice site favorability between D and D′ due a single nucleotide change (57). Two minor 5′-UTR splice variants were observed for *SFTPA1* (ACD′ and AB′D′) plus some rare (not shown in **Figure 1B**) variants (54). The transcripts from each gene carrying a different major or minor 5′-UTR splice variant are translated both *in vitro* (except for the AB′D′) (54) and *in vivo* as shown by polysome bound RNA (58). However, differences in both relative translatability and relative levels of the splice variants were observed among individuals (58).

Extensive sequence variability including small deletions/insertions was also observed in 3′-UTR (54, 56, 59, 60). This variability included an 11-nucleotide (11-nt) insertion/deletion (**Figure 1C**) that was initially described for an SP-A1 variant named 6A^1^ (59). This 6A^1^ variant was identical to the most frequently found *SFTPA1* variant, 6A^2^, except at a single nucleotide (54). However, since the 6A^1^ variation was not present in subsequent sequencing data, it was presumed that this was a sequencing error and the 6A^1^ is referred to as 6A^2^ thereafter. This 11nt sequence provides potential binding sites for miRNA (61), shown to regulate expression (62). In addition to the 11-nt, other elements have been identified, by sequence comparison, in the 3′-UTR of *SFTPA1* and *SFTPA2*. These include the minimum AU-rich element motif UUAUUUAUU shown elsewhere to mediate mRNA degradation (63). This is present in *SFTPA2* variants at position 926-935 but not in the *SFTPA1* variants (61).

## 2 Regulation of *SFTPA*

Under baseline conditions, *SFTPA* mRNA levels vary significantly among individuals (64) with a sixfold difference between high and low expression among individuals (57). The lack of correlation between total *SFTPA* mRNA levels and the *SFTPA1/SFTPA2* transcript ratio indicated that the levels in an individual may vary as a function of the *SFTPA* genotype, where the level of transcription and/or stability of mRNA may differ among variants (57). A variability in SP-A protein levels in bronchoalveolar lavage is observed among individuals (65–68) and during development (69). Together these indicate that there may be mechanisms that differentially affect the expression of the SP-A variants. In fact, in response to a variety of stimulatory or inhibitory regimens in fetal lung explants or in the human adenocarcinoma H441 cell line, the levels of human SP-A protein and/or mRNA change significantly (70–73). Also, SP-A levels may change in certain disease states (65–67, 74–76). Furthermore, inhibitory or stimulatory substances were shown to differentially affect the regulation of *SFTPA1* and *SFTPA2* mRNA in fetal lung explants or cell lines (46, 77–79). Although differences in protein and mRNA levels in health and in various disease states have been observed, to the best of our knowledge no comprehensive study has been done to correlate mRNA levels of each human *SFTPA* gene/variant and protein levels in samples from patients or healthy individuals or experimental systems where each transcript in its entirety is studied. We speculate that given the opportunity for complex regulation, as discussed in this review, it is probably unrealistic to think that a direct correlation of the overall mRNA and protein levels could exist without providing additional specific information. Such information may include reference to the specific genetic variant, the splice variant, the specific conditions, i.e., exposure to various insults including environmental stressors, and other. Experimental models of 5′-UTR or 3′-UTR regions of the SFTPA1/A2 variants are shown to exhibit differences in response to various insults and thus the mRNA/protein levels of the two genes and/or of their variants may differ. It would probably require additional reagents and approaches, such as gene- and variant-specific antibodies, and direct RNA sequencing to make such a correlation meaningful.

## 3 The Role of 5′ and 3′Untranslated Regions and Flanking Sequences in the Differential Regulation of *SFTPA1* and *SFTPA2* Transcripts

### 3.1 Impact of 5′-UTR Splice Variants in the Differential Regulation of *SFTPA1* and *SFTPA2*

These variants have been shown to regulate several steps/processes in SP-A regulation (80). They differentially affect translation and mRNA stability as assessed by *in vitro* transient expression of reporter gene constructs containing different 5′-UTR (A′D′, ABD, AB′D′ and A′CD′) splice variants. All variants compared to control vectors had a positive effect on gene expression as shown by increases in reporter gene activity and mRNA levels, with the ABD performing significantly better than the rest. In terms of the translation efficiency index (reporter activity/mRNA) a differential effect was observed by the splice variants. Compared to the control, both ABD and ABD′ exhibited higher translation efficiency whereas the other two splice variants, A′D′ and A′CD′, exhibited a lower efficiency. Algorithms predicting the secondary structure stability of the 5′-UTRs revealed that, compared to others (A′D′, AB′D′, A′CD′), the ABD structure was the most energetically favored one. Furthermore, the ABD was shown to exhibit a lower rate of mRNA decay upon inhibition of transcription with actinomycin D. Collectively, these indicate that the ABD splice variant has a better secondary structure stability and a lower rate of mRNA decay (80).

Splice variants (ABD, A′D′, A′B′D with the exception of A′CD′) were shown to differentially mediate internal ribosome entry site (IRES) activity i.e., cap-independent translation with the ABD exhibiting the highest IRES activity and A′D′ the next highest whereas the AB′D′ and A′CD′ exhibited low or no IRES (81). Secondary structure stability and especially the presence of a double loop structure in ABD and A′D′ (but absent in AB′D′ and A′CD′) as well as cis acting elements (in ABD) and perhaps other factors may all differentially contribute to the cap-independent translation. The ABD IRES activity was responsive to specific environmental stressors (i.e., to diesel PM but not to ozone exposure). Furthermore, the double-loop structure, which is important in cap-independent translation, didn’t seem to be necessary for cap-dependent translation activity, as shown with the A′D′ splice variant (81).

One major difference between the major *SFTPA1* and *SFTPA2* 5′-UTR splice variants is the presence or absence of exon B (eB). The eB presence in the UTR, as discussed above, results in a better outcome, whether mRNA stability, rate of mRNA decay, secondary structure and perhaps other, indicating that eB may be an important regulatory element. In a series of reporter gene constructs or *in vitro* translation experiments, eB was found to be an enhancer of transcription, if placed upstream of heterologous 5′-UTR or in its natural 5′-UTR, as it increased mRNA content regardless of position or orientation (82). eB also increased translation of mRNA reporter transcripts in the presence or absence of poly-A, when placed within its natural sequence environment but in heterologous 5′-UTR increased translation only in the presence of poly-A (82).

**Figure 2:**
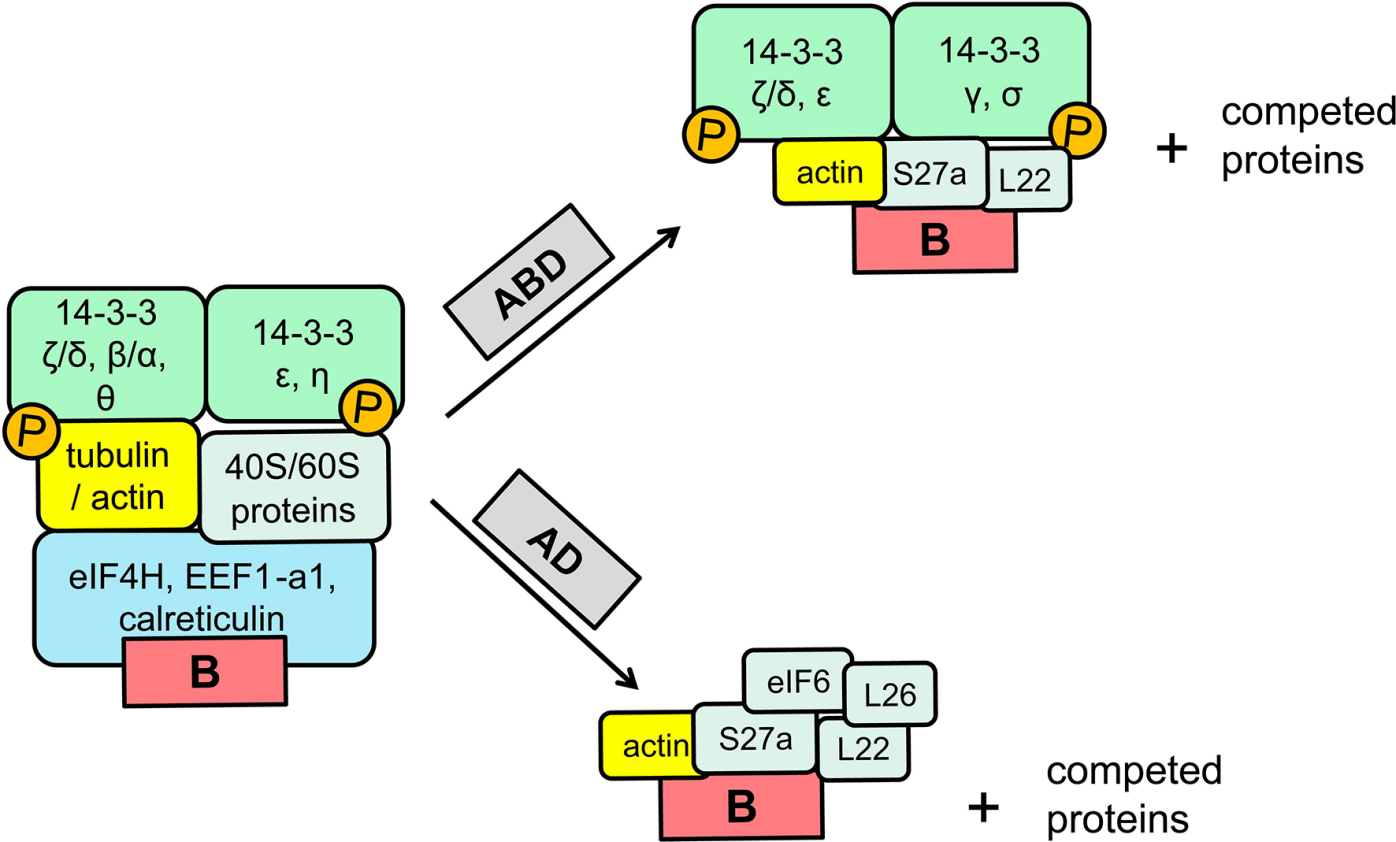
Schematic representation of the proteins present in the eB-mediated shifts before and after competition with excess of ABD or AD RNAs [adapted from Noutsios et al. (87)]. The identity of the proteins present in the shifts before and after competition were identified with mass spectroscopy, as described in detail elsewhere (86, 87).

The 14-3-3 proteins form homo- or heterodimers and by binding a variety of ligands including kinases, phosphatases, transmembrane receptors, etc., regulate a variety of functions, including cell cycle control, translation, apoptotic cell death, other (83–85). eB interacts either alone or within the context of the surrounding 5′-UTR sequences with 14-3-3 proteins. RNA pulldown assays, RNA affinity chromatography and surface plasmon resonance analyses showed that eB binds directly most of the 14-3-3 protein isoforms (β, γ, ϵ, η, σ, τ/θ) except isoform zeta (ζ). The latter isoform may bind eB indirectly because isoform zeta was identified by mass spectroscopy of shift and pull-down assays to be part of the eB-protein complex. Regardless of its presence in the eB-protein complex isoform zeta does not affect SP-A2 levels upon inhibition by shRNA knocked down (86). However, inhibition of the other 14-3-3 eB binding isoforms resulted (except isoform σ) in a downregulation of SP-A2 without any change in SP-A1 levels. Isoform σ did not show any gene-specific downregulation, as the levels of both SP-A1 and SP-A2 were negatively affected. Furthermore, differences in the stability of eB/14-3-3 isoform complexes have been observed (86). Deletion and mutation mapping analyses revealed two regulatory motifs in eB, GUCGCUGAU (next to exon A) and GGAGCCUGAA (near exon D) that are important for protein binding as assessed by shift assays (87). The eB RNA/protein complexes, one major and one minor contain in addition to the 14-3-3 proteins a number of other proteins that include, among others, ribosomal, cytoskeletal and translation factor proteins (87). Competition experiments with excess AD or ABD RNA of the eB-mediated shifts did not disrupt the eB shifts entirely (as the eB RNA competitor did) but resulted in altered mobility shifts with a lower size. The collective observations of the eB shifts competed with AD or ABD excess RNAs along with the mass spectroscopy data of the identity of the proteins in the eB-shifts before and after competition are summarized in a schematic representation of **Figure 2** [adapted from (87)]. The 14-3-3 proteins surprisingly were not competed with the ABD RNA but these were indeed competed with the AD RNA competitor. The reasons for this are not clear. The 14-3-3 proteins were present in shifts with either the eB or ABD probe but not with the AD. It was postulated that since in silico analysis showed that 6nt at the 3′end of exon A were part of an eB regulatory element, one possibility is that the ABD but not the AD provided some kind of stability at the junction of A-B resulting in a partial displacement.

**Figure 3:**
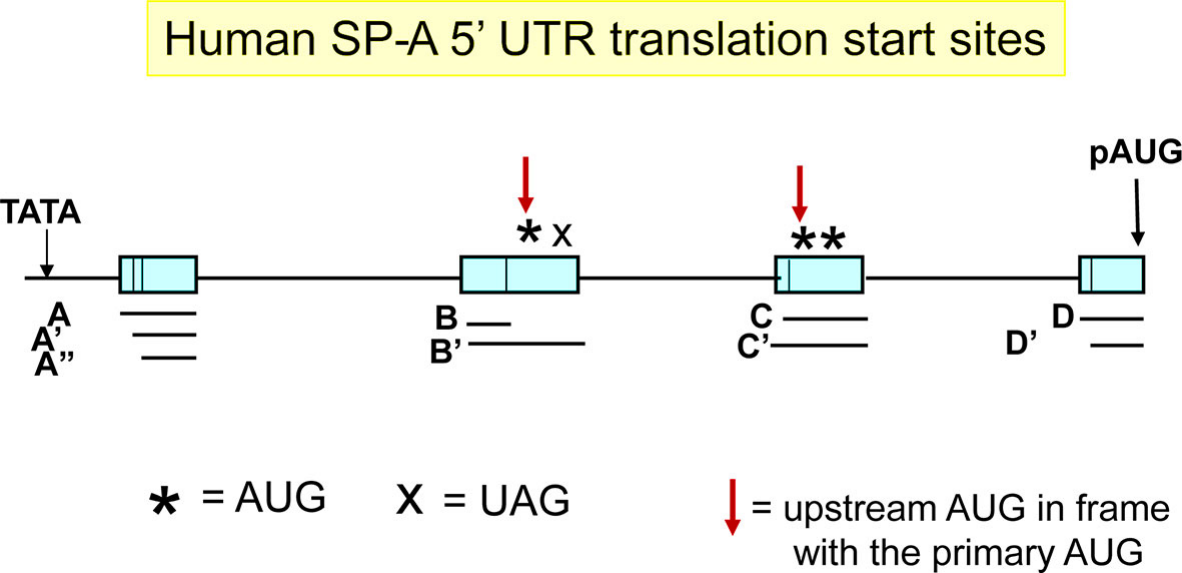
Translation start sites at the 5′-UTR. The primary translation start site (pAUG) is marked with a black arrow. Other upstream translation start sites (uAUG) in frame with the pAUG are marked with a red arrow. X denotes a stop codon in frame with the immediately uAUG.

The ACD′ 5′ UTR splice variant has been described as a minor splice variant of SP-A1 transcripts and is found only in SP-A1 transcripts (54, 56). The exon C of this splice variant is 60 nucleotides long and contains two upstream AUG (uAUG) sites in addition to the primary (p) AUG (**Figure 3**). The AUG closer to the TATA box is in frame with the pAUG whereas the other one is not. The in-frame uAUG results in an N-terminally extended isoform, but the additional residues do not seem to alter the site of cleavage by the signal peptidase (88). The out-of-frame uAUG introduces an ORF that overlaps with the primary ORF (the stop codon is within the coding region of *SFTPA1*, corresponding to residues 72-73 of the protein product of the main ORF). The production of any peptide from the overlapping ORF and its effects (cis or trans) on SP-A1 protein production have yet to be evaluated. An uAUG has also been described for exon B′ (**Figure 3**). However, this uAUG, although in frame with the pAUG, is followed with an in-frame stop codon, eight nucleotides downstream. Using a variety of approaches, Tsotakos and colleagues (88) showed that the uAUGs in the ACD′ splice variant decrease SP-A1 expression without affecting the size of the mature protein. The ACD′ transcripts appear to be present in the majority of individuals and their expression can be affected by mechanical injury. Their contribution to the SP-A1 transcript pool may be regulated by different stimuli including LPS and dexamethasone. Moreover, the SP-A1 AD′ (major) 5′ splice variant and the SP-A1 ACD′ (minor) 5′-UTR variant may be differentially regulated (88). Interestingly, the presence of exons C or B′ in the ACD′ and AB′D′ splice variants of the *SFTPA1* transcript, respectively, may introduce G-guadruplex structures in the 5′-UTR, which may affect translation initiation (89, 90). Such structures are absent from all *SFTPA2* splice variants, as analyzed by the online QGRS Mapper tool (91).

There are five polymorphisms in the 5′-UTR of *SFTPA1* that are categorized as possibly loss of function (pLoF) by the Genome Aggregation Database (gnomAD, v.3.1.1) (92), out of a total of 271. Specifically, rs1317624468 and rs1020324172 may affect splicing of the 5′-UTR exons, but the frequency of either variant is very low. This means that there is low confidence in these variants. Similarly, in the 5′-UTR of *SFTPA2*, there are 12 pLoF out of a total of 290 polymorphisms. As is the case with variants in *SFTPA1*, the identified short nucleotide variants (SNVs) seem to be associated with splice donor/acceptor sites, but the confidence on the effect these variants have is low, given their low frequencies. The SNVs were identified with the use of the UCSC Genome Browser (93).

### 3.2 3′-UTR-Mediated Regulation of Human *SFTPAs*

A number of *SFTPA1* and *SFTPA2* transcripts have been identified. These encode different protein variants i.e., with differences in their coding region, and these protein variants are discussed elsewhere (1). The 3′-UTRs of the transcripts of these protein variants, in transient transfection experiments compared to control vector, have been shown to differentially reduce mRNA and protein levels, as assessed by the activity of the reporter gene at baseline and in response to dexamethasone treatment (94). The inhibition in response to dexamethasone is glucocorticoid specific as both dexamethasone and hydrocortisone decreased reporter gene activity (95). Dihydrocortisone and phorbol ester 12-O-tetradecanoylphorbol-13-acetate on the other hand did not have any effect on reporter gene activity. The former was shown previously not to regulate SP-A (47) and the latter to affect SP-A regulation at the transcription level (96).

The 3′-UTRs of the *SFTPA1* transcripts encoding protein variants, 6A^2^, 6A^3^, 6A^4^, exhibit a differential effect on translation with no significant difference found between the 3′-UTRs of the two studied *SFTPA2* protein variants, 1A^0^ and 1A^3^ (61). An 11-nt element, described in the introduction (59), is located at position 405. This element that is present in the 3′-UTR of all the *SFTPA2* 3′-UTR sequences investigated to date and in the *SFTPA1* transcript encoding the 6A^2^ protein variant but absent in other *SFTPA1* transcripts studied, had a negative impact on translation. Upon its removal, translation increased, and the stability of the predicted secondary structure was changed. *In silico* analysis of the 11-nt element revealed seven potential miRNA binding sites (61). miRNAs are small noncoding RNAs that regulate gene expression at the posttranscriptional level *via* interactions with untranslated mRNA sequences.

Three miRNAs (miRNA-183, miRNA-4495 and miRNA-612) with potential binding sites within or near the 11-nt sequence (**Figure 1C**), *via* the use of miRNA mimics and/or antagomirs, were shown to inhibit gene expression of all 3′-UTR- constructs that included the 11-nt element (i.e., all *SFTPA2* transcripts and the *SFTPA1* transcript of the 6A^2^ protein variant (62). One miRNA (miRNA-4507) negatively affected the reporter gene activity of *SFTPA1* transcripts that lacked the 11-nt sequence, and another (miRNA-767) inhibited expression of both *SFTPA1* and *SFTPA2* transcripts. Collectively, these data indicate that miRNA regulatory pathways are involved in the SP-A regulation. This has been further validated with the knockdown of Drosha, an important effector of miRNA maturation. Inhibition of Drosha in primary human alveolar type II cells *via* siRNA, resulted in an increase in the levels of SP-A (97).

In summary, both 5′-UTR and 3′-UTR are important in the regulation of the human SFTPAs. Transient transfection experiments of reporter genes showed that 5′-UTR and 3′-UTR have an additive effect on translation. In addition, the poly-A tail also contributes to *SFTPA* regulation (61). Transcripts of constructs containing *SFTPA2* 5′-UTR variants in the presence or absence of poly-A, displayed a higher level of *in vitro* translation products than *SFTPA1* 5′-UTR (AD′). Moreover, the presence of the poly-A tail, even in the absence of 3′-UTR, increased translation (61).

### 3.3 SP-A Flanking Sequences in the Regulation of SP-A1 and SP-A2

The 5′-flanking regions of the *SFTPA* genes have been studied in multiple species. Sequence comparisons between rat and human *SFTPA* genes identified one proximal (up to 225bp upstream of TSS) and one distal (-1115bp in rats/-938bp in humans) conserved element in the 5′-flanking regions (98). This conservation led to further exploration of the flanking regions for regulatory elements. Sequencing analysis of these regions in rat, rabbit, baboon and human (19, 21, 98, 99) led, as described below, to the identification of several cis elements. These elements act as binding sites for transcription factors during both basal expression and in response to cellular signals.

#### 3.3.1 Promoter Analysis in Animal Species

Developmental studies using rabbit and baboon as model identified a DNAse I-hypersensitivity site -180 to -80 bp of the TSS after gestational day 21 in rabbits and day 140-160 in baboons, indicating potential changes in the proximal promoter region around the developmental timing of gene activation (100). Primer extension analysis of the upstream sequence in rabbits revealed an octamer that is one nucleotide different from the consensus cAMP response element (CRE) at -261bp (100). Interestingly, this CRESP-A fails to bind the CREB transcription factor or a basic leucine zipper polypeptide, indicating that a different transcription factor may be responsible for binding to this specific site (101). Similar studies followed the characterization of the two *SFTPA* genes in baboons (19). Regulation patterns in the presence of dibutyryl-cAMP and dexamethasone in the baboon were similar to the ones observed in the rabbit model (102) prompting further study of the flanking sequences.

**Figure 4:**
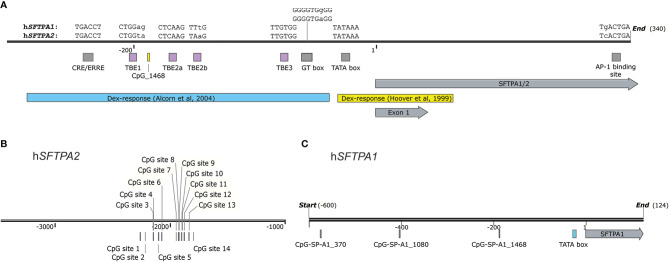
**(A)** A map of the promoter region of the human SP-A genes (-300 to +340) drawn to scale. Elements in a yellow box are studied in h*SFTPA1* and the ones in blue boxes in h*SFTPA2*. Grey boxes indicate regulatory elements found in both genes. The differences in sequences are demarcated by lowercase letters. The dexamethasone response elements are based on the transfection studies by Hoover et al. with h*SFTPA1* promoter constructs in H441 cells (45) and by Alcorn et al. with h*SFTPA2* constructs in A549 cells (110). **(B)** The relative positions of the methylation island identified in an enhancer region of h*SFTPA2* is shown (49). **(C)** The relative positions of CpG sites upstream of the *hSFTPA1* gene is according to Lin et al. (48).

Analysis of the rat sequences and CAT reporter assays identified a silencing element between base pairs -195 and -163, which was bound by members of the C/EBP family of transcription factors (103, 104). Further analysis of the rabbit SP-A promoter by fusion of different promoter sites with the human growth hormone (hGH) structural gene, used as reporter, revealed potential binding sites for several transcription factors, such as Sp1 at -190bp and AP-1 at -416 and -255bp. Four elements homologous to glucocorticoid response element (GRE) were also identified (99). In addition, two E-box sequences, one proximal and one distal, were identified. These seem to be bound by homo- and heterodimers of the Upstream Stimulatory Factors 1 and 2 (USF1 and USF2), playing a role in basal and hormonal regulation of the rabbit *SFTPA* gene (105, 106). An E-box motif was also identified in positions -8 to -3 of the murine SP-A gene promoter, but it was considered to not play a lung-specific regulatory role as assessed by transfection of MLE-15 cells, a cell line derived from lung tumors produced in transgenic mice expressing SV40 large T antigen driven by the lung-specific human SP-C promoter (107). Furthermore, DNAse I fingerprinting assays and EMSA with bacterially expressed TTF-1/Nkx2.1 revealed three binding sites that comprise a TTF1-binding element (TBE) for each baboon gene (108). A similar element with four binding sites was discovered in the murine *SFTPA* promoter (107). In the rat *SFTPA* gene promoter, there is an insertion in positions -316 to -211 that is considered to have occurred after the divergence of the mouse and rat lineages. Within the conserved sequences, there are five potential TTF-1 binding sites, of which at least four were present in protected regions by DNAse I fingerprinting analysis (55, 109). The glucocorticoid inhibition of SP-A expression was found to be mediated by TTF-1 (110). The activity of TTF-1 is dependent on phosphorylation by Protein Kinase A (PKA), a cAMP-induced kinase (111). During development, TTF-1 expression depends on the presence of certain microRNAs (112) and the Hepatocyte Nuclear Factor 3β (HNF3-β). The latter belongs to the winged family of transcription factors and targets other genes critical for the differentiation of respiratory epithelial cells (113). PKA activated by cAMP increases TTF-1 phosphorylation and binding to the TBE (111) but also enhances the interaction of TTF-1 with the CREB-interacting protein (CBP) and the steroid receptor coactivator 1 (SRC-1, a different member of the nuclear receptor coactivator family) (114). The TBE also contains a reverse-oriented NF-κB binding site (115). Interleukin-1 treatment, along with TTF-1, increases NF-κB binding to the TBE (115). Dexamethasone increases expression of the NF-κB inhibitor, IκB-α, thus blocking its transcriptional activity (110), while increasing recruitment of histone deacetylases 1 and 2 near the TBE (**Figure 4A**, highlighted by the blue ribbon), as shown by chromatin immunoprecipitation (116). NF-kB seems to be the common mediator of the developmental timing of expression of genes that are involved in lung innate immunity and surfactant homeostasis, as indicated by time-dependent transcriptome profiling in two strains of mice (117). Two more factors co-regulate SP-A expression along with TTF-1, at least in mice; GATA-6, which binds to a GATA-binding site at positions -69 to -64 of the murine gene promoter (118) and B-Myb, which binds to an element in positions -380 to -371 (119). High throughput ChIP-seq analysis of TTF-1/Nkx2.1 confirmed its binding to genes critical for lung function and health (120, 121). Although a TBE containing three TTF-1 binding sites was also identified in the h*SFTPA2* as a cAMP-responsive cis element in studies using transgenic mice, only one of the binding sites (the middle one) is identical between the baboon and human *SFTPA2* (122).

#### 3.3.2 *SFTPA1* and *SFTPA2*

Hormonal regulation studies by transfection of lung cell lines of human origin with WT and CRE-mutated constructs verified the responsiveness of the CRE to the cAMP analogue dibutyryl-cAMP (Bt_2_cAMP) (99). Dexamethasone inhibits the cAMP-induced expression of the reporter gene despite stimulating transcription of *SFTPA* in the absence of cAMP in the same system, indicating an interaction between the CRE and a glucocorticoid response element. Critically, the two human genes are differentially regulated by a number of signaling molecules, such as cAMP, glucocorticoids, and insulin (46, 77, 79), a fact that highlights the importance of the promoters as elements that mediate differential expression of the two genes. Such studies are further complicated by the fact that the dexamethasone effect in SP-A gene expression is biphasic and dose-dependent (47, 70, 123).

Subsequent studies focused mostly on regulatory elements of *hSFTPA2*, as it was found to be more responsive to stimulatory effects by cAMP than *hSFTPA1* (79). Studies using transfected type II cells identified a CRE element in the *hSFTPA2* gene (124), although similar studies have not been performed for the *SFTPA1* gene. The CRE element is bound by the Estrogen-Related Receptors, ERRα and ERRγ, but only the ERRα receptor increased h*SFTPA* transcription, while the ERRγ had no effect (125). A mechanistic study confirmed that the action of ERRα is mediated by PKA and revealed SRC-2 (steroid receptor coactivator 2) as a cofactor of the cAMP-induced transcriptional activation of SP-A (126). SRC-2 is downregulated by the glucocorticoid receptor (127), thus partially explaining the dexamethasone-induced mitigation of cAMP-dependent *SFTPA2* transcription. Moreover, the CRE (or ERRE, Estrogen-Related Receptor Element, **Figure 4A**) works cooperatively with a GT box (128) to mediate basal and cAMP-induced changes in SP-A2 expression. Based on supershift experiments, the GT box is bound by at least five protein complexes, two of which contain Sp1, a ubiquitously expressed transcription factor (128).

With regards to inhibitory stimuli, h*SFTPA1* was found to be more responsive to dexamethasone treatment than h*SFTPA2* in H441 cells (78, 79) and in fetal lung explants treated with 100nM dexamethasone, the expression of *SFTPA1* mRNA was inhibited to a greater degree than *SFTPA2* (77). Study of the mechanistic aspects of this inhibition identified the -32/+63 region (relative to the TSS) as the dexamethasone response element, indicated by a yellow band in **Figure 4A** (45). Removal of the region -227/-31, encompassing the CRE/ERRE, GT box, and TBE (111, 124, 128), did not affect the dexamethasone response, but it significantly attenuated the basal transcriptional *hSFTPA1* promoter activity, indicating that these elements are not involved with this dexamethasone-mediated regulatory pathway (45). Another inhibitory agent for both human genes is phorbol ester (96). Deletion analysis to identify promoter elements that are responsible for this effect identified a region downstream of the TTS (+309/+329 in *hSFTPA1*). A member of the Jun, but not of the Fos family of proteins, was identified by supershift assays as a binding transcription factor to this site (129). Furthermore, the +318/+324 region contains a sequence (TGACTGA) similar to the AP-1 consensus binding site (shown as “AP-1 binding site” in **Figure 4A**), thus implicating AP-1 complexes in the transcription of the *SFTPA* genes.

A functional study employing CRISPRi, a method using a fusion of deactivated Cas9 and the repressor KRAB, thereby inhibiting genes without deleting them, was performed on identified targets of TTF-1/Nkx2.1, including *SFTPA1* and *SFTPA2* (130). Prior ChIP-seq analysis had indicated that TTF-1 binds to a proximal upstream region of *SFTPA1* and a distal upstream region of both *SFTPA1* and *SFTPA2* (131, 132). Targeting either of these sites with CRISPRi in A549 and H441 cells suppressed the expression of *SFTPA1* significantly, but *SFTPA2* was suppressed only by the sgRNA targeting the distal region (130). Deletion of the distal region, which is about 20kbp away from either gene’s TSS and is located close to the *SFTPA3P* pseudogene, by the CRISPR/Cas9 approach in A549 repressed the expression of both *SFTPA1* and *SFTPA2* (130), which may indicate a regulatory role of the pseudogene.

The presence of two surfactant protein genes with high degree of similarity in the flanking regions makes it technically challenging to discern mechanisms of differential regulation. The combination of models, such as cell lines and animal models has led however to the identification of several transcription factor binding sites (**Figure 4**). At the very least, ERRα, USF1/2 heterodimers, Sp1, TTF-1/Nkx2.1 and AP-1 complexes have been shown to regulate SP-A transcription, but any differential effects have yet to be shown unequivocally. Advances in gene editing techniques may elucidate this topic in the future.

## 4 Epigenetic Regulation

Alterations of *SFTPA* in lung cancer have been observed in various experimental settings (133–139). In a few cases, *SFTPA* was shown to be useful as a marker of differential diagnosis of metastatic lung cancer and mesotheliomas (138, 140–143). A high-throughput approach identified aberrant methylation of CpG sites of several genes to associate with lung cancer (144) indicating that this may be a contributing process in lung cancer. A subsequent DNA CpG methylation profiling of the *SFTPA1* gene promoter identified two CpG *SFTPA1* sites (*SFTPA1*_370 and *SFTPA1*_1080, **Figure 4C**) to be hypomethylated in lung cancer (adenocarcinoma and squamous cell carcinoma). In normal lung tissue the level of methylation of another *SFTPA1*_1468 CpG site (not shown to significantly differ in its methylation content in cancer lung tissue) was associated with the level of *SFTPA1* transcripts. This CpG is located 160 nucleotides upstream of the TATAA box. The high level of unmethylated CpG_1468 was correlated with a high level of *SFTPA1* transcripts indicating that the methylation status of this site may play a role in SP-A1 expression. This site is absent from *SFTPA2*. Of relevance, rare *SFTPA1* transcripts, including a more frequently found *SFTPA1* transcript coding for the 6A^4^ protein variant, were shown to associate with risk for lung cancer (145). A CpG DNA site methylation difference was also observed between normal and cancer lung tissue for the *SFTPA2* gene promoter (49). This CpG site is located -2215 upstream of the transcription start site (**Figure 4B**) and exhibited a higher level of methylation in lung cancer especially in adenocarcinoma. Moreover, the level of *SFTPA2* mRNA and protein were reduced in lung cancer whereas the mRNA level of DNA methyltransferases (DNMT1 and DNMT2) was increased. An *in-silico* analysis revealed a number of potential binding sites of transcription factors around this CpG methylation site indicating that apart from its potential as a marker, this CpG site modification may interfere with the binding of regulatory factors affecting *SFTPA2* expression.

DNA methylation, which is affected by environmental factors, air pollution, smoking, diet among others (146–150), may be a regulatory mechanism for *SFTPA* gene expression. The differential regulation of the methylation status of specific CpGs in *SFTPA1* and *SFTPA2* maybe one of the epigenetic processes that does not only apply to lung cancer but also to other health states (151). Furthermore, epigenetic phenomena that modulate changes in gene function without changing the nucleotide sequence include several processes, such as DNA methylation, histone modifications, miRNAs, and splice variants. The role of miRNAs, splice variants and DNA methylation has been discussed above. Histone acetylation and methylation have been shown to affect *SFTPA* expression in the lung during development and hypoxia (50, 116, 152). The developmental timing of *SFTPA1* expression is associated with enhanced acetylation and decreased methylation of histone H3 at the *SFTPA* promoter. Histone methyltransferases Suv39H1 and Suv39H2 are bound to the TBE prior to induction of *SFTPA* by cAMP (152). Their transcript levels are inversely correlated with the developmental pattern of SP-A expression (152). Increased O_2_ tension facilitates the induction of histone H3 acetylation on lysines 9 and 14 at the TBE, while hypoxia induces dimethylation of lysine 9 of H3 (50) and recruitment of Suv39H1 and Suv39H3 to the TBE (152). Dexamethasone treatment increased nuclear levels of the histone deacetylases HDAC-1 and HDAC-2, but not the total levels of histone H3, in human fetal type II cells (116). ChIP analysis indicated that dexamethasone also increases the occupancy of the TBE specifically by HDAC-1 and HDAC-2. The cAMP analogue Bt_2_cAMP increased the levels of acetylated and phosphorylated histone H3; this effect was antagonized by dexamethasone, which promoted demethylation of H3K9 globally and locally, in the TBE region of the *SFTPA* promoter (116).

Although differential allele expression has not been studied for the human *SFTPA* genes either in the lung or extrapulmonary tissues, it is worth noting that this mechanism may be operative under certain conditions, as it is shown for the rat *SFTPA* under unperturbed conditions (153). *SFTPA* was shown under these conditions to exhibit a balanced biallelic expression in the lung but in colon was both balanced and imbalanced and family studies indicated that inheritable factor(s) may contribute to the regulation of differential allele expression (153).

## 5 Conclusion/Discussion

Understanding differences between human *SFTPA1* and *SFTPA2* genes and their corresponding variants is both useful and important as such information could be implemented in personalized regimens. For example, in a pilot study where precision cut lung slices from human donor lungs were treated with varying concentrations of methylprednisolone, a pharmacologic relationship was observed between treatment and *SFTPA* genotype (154). As lung donors and recipients are treated with immunosuppressive regimens, one may in future pharmacogenetic studies of lung transplants further investigate as well as consider immunosuppressive regimes tailored according to the donor’s genetic background. Recent developments in sequencing technologies, particularly the advent of long-read sequencing, could contribute to our understanding of variant frequencies and their potential regulation. Furthermore, identification of key regulators, either cis or trans, can be useful in modulating specific *SFTPA1* or *SFTPA2* gene expression as it may be appropriate i.e., in the case of the prematurely born infants where levels of SP-A are low and/or in the course of infection. Of interest, the *SFTPA2* transcript coding for the 1A^0^ protein variant has been shown to exhibit a protective effect in terms of survival in both animal models after infection and/or other insults (155, 156) and in lung transplant patients (39). Thus, in such cases it could be advantageous to modulate expression of SP-A1 and/or SP-A2 proteins.

## 6 Outstanding Issues and Future Studies

The work presented in this review indicates that the human *SFTPA* genes are under extensive and complex regulatory control that merits further experimentation. Our current knowledge on this topic is based for the most part on studies where different regions of a given gene was studied i.e., flanking region, 5′-UTR, 3′-UTR. So at the present time it is difficult to know exactly how a given variant will respond to a stimulus or under a certain environmental condition in its entirety. For example, regulatory mechanisms of one region (i.e., 5′-UTR) may attenuate or enhance i.e., an up or down regulation imparted by mechanisms operative in another region (i.e., 3′-UTR, flanking region) or even nullify effects. A major advance to our understanding could be achieved if the entire *SFTPA* locus is studied as a unit in order to better assess the commonalities and differences between the two genes as well as the potential role of the *SFTPA3P* on the regulation of the functional genes, as pseudogenes may contribute to the regulation of their functional counterpart (157–159). It is currently unknown whether *SFTPA3P* is expressed or not, and if so, under what conditions and at what developmental stage. If it is expressed, it could act as a lincRNA, thus regulating the neighboring parental genes in a number of possible ways (160). Given that the two functional genes are in opposite transcriptional orientation and may share regulatory cis elements that may or may not work in a coordinated regulation, examining the entire locus as a whole would be a sensible approach, albeit one with substantial challenges. Identifying an appropriate study system would be a start. Given the size of the locus, generation of humanized mice would be technically challenging, so studies in non-human primates might be more appropriate.

## Author Contributions

JF had the idea for the article. JF and NT performed the literature search and wrote the manuscript. All authors contributed to the article and approved the submitted version.

## Conflict of Interest

The authors declare that the research was conducted in the absence of any commercial or financial relationships that could be construed as a potential conflict of interest.

## Publisher’s Note

All claims expressed in this article are solely those of the authors and do not necessarily represent those of their affiliated organizations, or those of the publisher, the editors and the reviewers. Any product that may be evaluated in this article, or claim that may be made by its manufacturer, is not guaranteed or endorsed by the publisher.
